# A biologically constrained spiking neural network model of the primate basal ganglia with overlapping pathways exhibits action selection

**DOI:** 10.1111/ejn.14869

**Published:** 2020-07-03

**Authors:** Benoît Girard, Jean Lienard, Carlos Enrique Gutierrez, Bruno Delord, Kenji Doya

**Affiliations:** ^1^ Institut des Systèmes Intelligent et de Robotique (ISIR) Sorbonne Université CNRS Paris France; ^2^ Neural Computation Unit Okinawa Institute of Science and Technology Kunigami‐gun Japan

**Keywords:** action selection, basal ganglia, centromedian/parafascicular thalamus, computational model, monkey

## Abstract

Action selection has been hypothesized to be a key function of the basal ganglia, yet the nuclei involved, their interactions and the importance of the direct/indirect pathway segregation in such process remain debated. Here, we design a spiking computational model of the monkey basal ganglia derived from a previously published population model, initially parameterized to reproduce electrophysiological activity at rest and to embody as much quantitative anatomical data as possible. As a particular feature, both models exhibit the strong overlap between the direct and indirect pathways that has been documented in non‐human primates. Here, we first show how the translation from a population to an individual neuron model was achieved, with the addition of a minimal number of parameters. We then show that our model performs action selection, even though it was built without any assumption on the activity carried out during behaviour. We investigate the mechanisms of this selection through circuit disruptions and found an instrumental role of the off‐centre/on‐surround structure of the MSN‐STN‐GPi circuit, as well as of the MSN‐MSN and FSI‐MSN projections. This validates their potency in enabling selection. We finally study the pervasive centromedian and parafascicular thalamic inputs that reach all basal ganglia nuclei and whose influence is therefore difficult to anticipate. Our model predicts that these inputs modulate the responsiveness of action selection, making them a candidate for the regulation of the speed–accuracy trade‐off during decision‐making.

## Graphical abstract

We designed a spiking model of the basal ganglia of the monkey, derived from a mean‐field function‐agnostic model. Following monkey neuroanatomic data, it is characterized by strongly overlapping direct and indirect pathways. We show action selection is an emerging property of the resulting system and that it does not depend on direct/indirect pathway segregation.

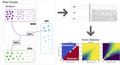

**Abbreviations**
AMPA: α‐amino‐3‐hydroxy‐5‐methyl‐4‐isoxazolepropionic acid
CM/Pf: centromedian and parafascicular thalamic nuclei
CSN: cortico‐striatal neurons
FSI: fast‐spiking interneurons
GABA: γ‐aminobutyric acid
GPe: external globus pallidus
GPi: internal globus pallidus
LIF: leaky integrate‐and‐fire neuron model
MSN: medium spiny neurons
NMDA: N‐methyl‐d‐aspartic acid
PSP: post‐synaptic potential
PTN: pyramidal tract neurons
STN: subthalamic nucleus

## 1 INTRODUCTION

**FIGURE 1:**
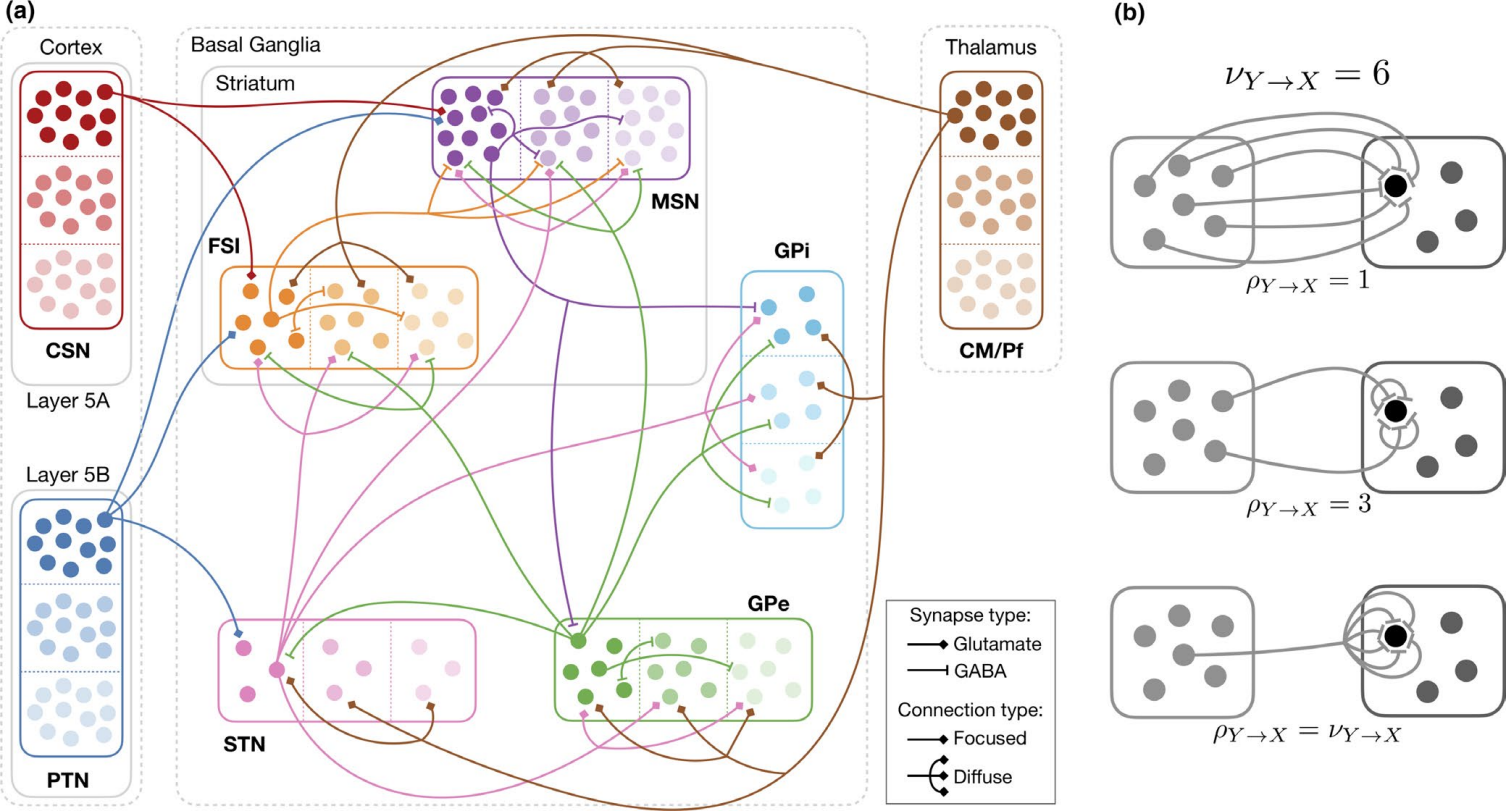
Structure of the basal ganglia model. (a) Wiring diagram: filled circles represent neurons. Each population is composed of channels (three shown here), represented by different shades and separated by dashed lines. For the sake of simplicity, the projections of one neuron of the first channel in each population are shown. The number of neurons shown here is not precisely to scale: for example, each channel of the striatum is simulated with 10,576 neurons and each channel of the GPi with 56 neurons (the exact number of simulated neurons per channel, in accordance with their ratio in the primate brain, is documented in Table 1). (b) Illustration of redundancy: for a fixed number of input synapses $\nu_{Y\to X}$, here equal to 6, redundancy *ρ* can vary from 1 (each input synapse comes from a different neuron of *Y*, top) to *ν* (all synapses come from the same neuron of *Y*, bottom). We use *ρ* = 3 everywhere (middle). All figure by Girard, Liénard & Delord (20202020); available under a CC‐BY4.0 licence (https://doi.org/10.6084/m9.figshare.12311564)

The basal ganglia are a set of subcortical interconnected nuclei, which are thought to play a major role in action selection (Mink, 1996; Redgrave, Prescott, & Gurney, 1999) and reinforcement learning (Houk, Adams, & Barto, 1995; Schultz, Dayan, & Montague, 1997) in vertebrates, but whose complex interconnection scheme is still not fully understood. In 1989, Albin, Young, and Penney (1989) proposed an interpretation of the basal ganglia circuitry aimed at explaining various motor disorders, including Parkinson's disease: the operation of the basal ganglia would result from the interactions of two segregated and opposing pathways. In this scheme, the *direct pathway* corresponds to focal inhibitory projections from the striatum to the output of the circuit. The *indirect pathway*, also originating in the striatum, is made of a cascade of inhibitions and excitations that have a net diffuse excitatory effect on the output nuclei. These two segregated pathways are supposed to stem from two distinct striatal neuron populations: one expressing receptors which are modulated by dopamine in an excitatory manner (D1 receptors) for the direct pathway, and the other one expressing receptors with an inhibitory modulation (D2 receptors) for the indirect pathway. The imbalance between these pathways, caused by alterations of the dopaminergic system, would explain the motor disorders. Although it neglects a large number of documented projections (Figure 1a), this proposal has the advantage of disentangling the complexity of the circuit and of proposing a simple unifying explanation to different motor disorders. Since 1989, the basal ganglia have been the subject of intense modelling activity, and probably, more than a hundred models have been published in the scientific literature.

A striking fact is that all the basal ganglia computational models posterior to Albin et al. (1989), from the earliest (Berns & Sejnowski, 1996) to the most recent (e.g. Baladron and Hamker (2015); Wei, Rubin, and Wang (2015); Mandali, Maithreye Rengaswamy, Chakravarthy, and Moustafa (2015); Berthet, Lindahl, Tully, Hellgren‐Kotaleski, and Lansner (2016); Caligiore, Mannella, and Baldassarre (2019)), are built on this central idea that the basal ganglia circuit is segregated in *direct* and *indirect* pathways. The existence of a strict segregation between the pathways is central in a number of theories and models. This is, for example, the case in the context of decision‐making, where the indirect pathway could play a role in the adaptive adjustment of the decision threshold in evidence accumulation models (Wei et al., 2015) or in the regulation of the exploration/exploitation trade‐off (Mandali et al., 2015). Major reinforcement learning models also rely on specific associations of the pathways to the association of the direct pathway (respectively, indirect pathway) with the learning of Go (respectively, NoGo) responses (Collins & Frank, 2014; Dunovan & Verstynen, 2016; Frank, Seeberger, & O’reilly, 2004) or variations of this schema where the hyperdirect pathway (from the cortex to the STN) brakes to prevent selection, and the indirect pathway controls the inhibition of common mistakes (Baladron & Hamker, 2015). It is also the case in a recent alternate theoretical proposal, suggesting that the basal ganglia are involved in the learning control of vigour rather, where the direct (resp. indirect) pathways then represent positive (resp. negative) adjustments of the movement parameters (Yttri & Dudman, 2016).

When considering non‐human primates, this interpretation raises questions, highlighted as the second “problem on the basal ganglia” by Nambu (2008): anatomical results acquired in the 90s in cynomolgus monkeys (Parent, Charara, & Pinault, 1995) and later confirmed in squirrel monkeys (Lévesque & Parent, 2005), showing that the pathways’ boundaries are quite blurred, as more than 80% of striatal neurons simultaneously project to nuclei supposed to belong to segregated pathways (namely, the GPe on the one hand and the GPi or the SNr on the other hand). In other species, recent results follow the same trend, showing that pathway segregation may not be that clear (Cazorla et al., 2014; Fujiyama et al., 2011; Kawaguchi, Wilson, & Emson, 1990; Schmitt, Eipert, Kettlitz, Leßmann, & Wree, 2016; Wu, Richard, & Parent, 2000). The segregated pathway model has probably reached the limits of its explanatory power (Calabresi, Picconi, Tozzi, Ghiglieri, & Di Filippo, 2014), as many species seem to be fully viable despite clear segregation. This motivates a revision of the theories of basal ganglia circuitry operation, through the exploration of computational models of the basal ganglia that would exhibit the properties that are expected from the circuit, despite major pathway overlap.

The conceptual and abstracted rate‐based neural network models excel at illustrating idealized functions of the brain. Their simplicity comes with greater tractability and easier analysis. However, such abstracted models remain very far from the actual substrate to the extent that they become hard to disprove—a characteristic that weakens their potential for predictions and ultimately limits their explanatory power. On the other end, detailed neural models thrive to stick as close as possible to biological details and thus create the conditions for a constructive back‐and‐forth dialogue with experimental neurobiology. This advantage comes at a price, though, as detailed models are more complicated to implement, parameterize and analyse. In particular, the additional degrees of freedom introduced with such models make their parameterization difficult or indeed impossible given the available experimental data. The most complex models may require the arbitrary hand‐tuning of parameters or the mix of experimental data obtained in different species (rodents and primates, usually), and by doing so, their veracity, or usefulness, becomes questionable.

The model of Liénard and Girard (2014) was designed as a compromise between mathematical degrees of freedom and available experimental data. Its parameters were optimized to fit to a large set of known biological constraints, ranging from anatomical data (such as bouton count derived from single‐axon tracing study) to electrophysiological recordings (such as the change of firing rate after injection of AMPA, NMDA or GABA_A_ antagonists). It relied on a mean‐field formalism that allies the simplicity and speed of rate‐coding models—enabling optimization of parameters—while still being anchored in neuronal physiology, with parameters expressed in physical units and directly related to experimental data. Importantly, it relied on basal ganglia activity at rest (in normal and pharmacological conditions) and thus made no hypothesis on the function of the circuit (what we called function‐agnostic). The model was later extended to add biologically plausible temporal dynamics (Liénard, Cos, & Girard, 2017) while still using the mean‐field formalism. This was done through the inclusion of realistic axonal delays and used to pinpoint potential origins of *β*‐band oscillations within the basal ganglia circuitry.

In this work, we develop a spiking model of the monkey basal ganglia that is based on the parameters of the function‐agnostic mean‐field model optimized in Liénard and Girard (2014) and on the temporal extension from Liénard et al. (2017). The methodology adopted for translation from population to individual neuron levels of modelling is simple and requires a minimal number of additional parameters. We show that the resulting spiking model parameterizations pass the same validation tests as the original rate‐based ones. We then study the response of the model to its three sources of inputs (cortico‐striatal neurons, pyramidal tract neurons and centromedian/parafascicular thalamic neurons), showing the specificity of each of them. This step allows to define reasonable input configurations that we use to extensively test, and validate, the ability of the models to perform action selection, despite their overlapping pathways. We explore the involvement in selection of a number of specific elements of the circuit architecture (lateral and feedforward inhibitions, off‐centre/on‐surround modules), using circuit disruptions. We finally identify a possible role of the centromedian and parafascicular thalamic inputs in globally adjusting the sensitivity of action selection.

## 2 MATERIALS AND METHODS

### 2.1 Integrate‐and‐fire model

We describe here the mathematical formalism of the leaky integrate‐and‐fire (LIF) network models developed in this study, with parameter values based on the optimized mean‐field models of Liénard and Girard (2014). In particular, the internuclei projection scheme was conserved (Figure 1a). As with the mean‐field model of Liénard and Girard (2014), the connection scheme used in our integrate‐and‐fire network includes projections reported in primate basal ganglia that are rarely modelled: the subthalamo‐striatal (Nakano et al., 1990; Nauta & Cole, 1978; Parent & Smith, 1987; Sato, Parent, Parent, Levesque, & Parent, 2000; Smith, Hazrati, & Parent, 1990) and pallido‐striatal projections (Beckstead, 1983; Kita, Tokuno, & Nambu, 1999; Sato, Lavallee, Lavallee, Levesque, & Parent, 2000; Spooren, Lynd‐Balta, Mitchell, & Haber, 1996).

The subthreshold dynamics of the leaky integrate‐and‐fire model is governed by Equation (1), where *τ*_m_ is the membrane time constant, *V* the neuron potential, *E*_r_ the resting threshold, *R*_m_ the membrane resistance and *I*_in_ the input current.

(1) $$\tau_{m}\frac{\text{dV}}{\text{dt}}\;\;=\;E_{r}\;‐\;V\;+\;R_{m}I_{\text{in}}$$

In our simulations, we first chose to shift all resting potentials *E*_r_ to 0, which does not affect model generality. Therefore, when *V* reaches the firing threshold *θ* (the value of which is shown in Table 2 for each simulated population), a spike is emitted and the potential of the neuron is reset to zero. Second, the mean‐field model proposed in Liénard and Girard (2014) integrated a simulation of the post‐synaptic potentials (PSP) evoked by incoming spikes, using *α*‐functions, and is expressed in mV. Thus, we kept the same formalism at the spiking level: the simulated synapses directly generate input potentials *V*_in_, rather than currents, leading to the following formulation:

(2) $$\tau_{m}\frac{\text{dV}}{\text{dt}}\;=\;‐\;V\;+\;V_{\text{in}}$$

The input potential, *V*_in_, includes a fixed tonic component (*V*_C_) as well as the PSPs induced by incoming spikes *s_j_* from all the input neurons *j*, depending on the neurotransmitter type *n* of each connection:

(3) $$V_{\text{in}}\left(t\right)\;=\;V_{C}+\sum_{j\in J}\gamma_{j}\rho_{j}\sum_{n,s}f_{\alpha }^{n}\left(t‐\left(t_{j}^{s}+\delta_{j}\right)\right)$$

where the change of potential is dependent on the time since the emission of spike *s* (at time $t_{j}^{s}$) plus the transmission delay $\delta_{j}$. The *α* functions ($f_{\alpha }^{n}$) model the dynamics of the post‐synaptic potential at one synaptic site, with different temporal dynamics and amplitude depending on the neurotransmitter type *n* (AMPA and NMDA for glutamatergic spikes and GABA_A_ for gabaergic spikes). The *ρ_j_* factor represents the number of synapses per input neuron that we call redundancy. The *γ* factor represents the dendritic attenuation of the PSPs.

To build the ensemble of input neurons *J*, we first consider the populations providing inputs, as defined by the circuit connectivity (Figure 1a). Note that when examining selection properties, each population of the model is subdivided in channels, representing the competing options (three such channels are represented in Figure 1a), we chose the size of these channels to be 1/5,000 the total size of each population (values provided in Table 1). With respects to channels, projections from one population to another can be focused (channel to channel) or diffuse (all to all). To determine how many neurons from a given population *Y* will provide inputs to a neuron in population *X*, we first compute *ν*_(_ *_Y_*_→_ *_X_*_)_, the number of total input synapses from *Y* to *X* as in Liénard and Girard (2014):

(4) $$\nu_{Y\to X}\;=\;\frac{P_{Y\to X}n_{Y}}{n_{X}}.\alpha_{Y\to X}$$

**TABLE 1:** Parameters of the integrate‐and‐fire model imported from the mean‐field model. These fixed parameters were derived from a literature survey in Liénard and Girard (2014)

Parameter	Symbol	Value	Ref
Neurons per channel			
MSN	n_MSN_	10,576	^AB^
FSI	n_FSI_	212	^AB^
STN	n_STN_	32	^C^
GPe	n_GPe_	100	^C^
GPi/SNr	n_GPi_	56	^C^
Firing rate at rest (Hz)			
CSN	$\phi_{\text{CSN}}$	2	^DE^
PTN	$\phi_{\text{PTN}}$	15	^DE^
CM/Pf	$\phi_{\text{CMPf}}$	4	^F^
PSP amplitudes (mV)			
AMPA	A_AMPA_	1	^G^
GABA_A_	$A_{{\text{GABA}}_{A}}$	0.25	^G^
NMDA	A_NMDA_	0.025	^G^
PSP half‐times (ms)			
AMPA	D_AMPA_	5	^H^
GABA_A_	$D_{{\text{GABA}}_{A}}$	5	^H^
NMDA	D_NMDA_	100	^H^
Dendritic Properties			
Membrane resistance (Ω cm^2^)	*R_m_*	20,000	^I^
Intracellular resistivity (Ω cm)	*R_i_*	200	^I^
Mean dendritic extent (µm)			
MSN	$l_{\text{MSN}}^{\max}$	619	^B^
FSI	$l_{\text{FSI}}^{\max}$	961	^B^
STN	$l_{\text{STN}}^{\max}$	750	^JK^
GPe	$l_{\text{GPe}}^{\max}$	865	^L^
GPi/SNr	$l_{\text{GPi}}^{\max}$	1,132	^L^
Mean dendritic diameter (µm)			
MSN	$d_{\text{MSN}}^{\max}$	1	^B^
FSI	$d_{\text{FSI}}^{\max}$	1.5	^B^
STN	$d_{\text{STN}}^{\max}$	1.5	^K^
GPe	$d_{\text{GPe}}^{\max}$	1.7	^L^
GPi/SNr	$d_{\text{GPi}}^{\max}$	1.2	^L^
% of projection neurons^a^			
MSN → GPi	*P*_MSN→GPi_	82%	^M^
STN → GPe	*P*_STN→GPe_	83%	^N^
STN → GPi/SNr	*P*_STN→GPi_	72%	^N^
STN → MSN	*P*_STN→MSN_	17%	^N^
STN → FSI	*P*_STN→FSI_	17%	^N^
GPe → GPe	*P*_GPe→GPe_	84%	^O^
GPe → GPi/SNr	*P*_GPe→GPi_	84%	^O^
GPe → MSN	*P*_GPe→MSN_	16%	^O^
GPe → FSI	*P*_GPe→FSI_	16%	^O^

^A^: (Johnston, Gerfen, Haber, & van der Kooy, 1990) ^B^: (Yelnik, Francis, Percheron, & Tandéa, 1991) ^C^: (Hardman et al., 2002) ^D^: (Bauswein et al., 1989) ^E^: (Turner & DeLong, 2000) ^F^: (Matsumoto, Minamimoto, Graybiel, & Kimura, 2001) ^G^: (Liénard & Girard, 2014) ^H^: (Destexhe, Mainen, & Sejnowski, 1998) ^I^: (Koch, 2005) ^J^: (Rafols & Fox, 1976) ^K^: (Yelnik & Percheron, 1979) ^L^: (Mouchet & Yelnik, 2004) ^M^: (Lévesque & Parent, 2005) ^N^: (Sato, Parent, et al., 2000) ^O^: (Sato, Lavallee, et al., 2000).

^a^ The projections not reported in the table have a probability of 100%.

where *n_X_* and *n_Y_* represent the number of neurons in populations *X* and *Y*, *α*_(_ *_Y_*_→_ *_X_*_)_ the average number of synapses each neuron of *Y* makes in population *X,* and $P_{Y\to X}$ the proportion of neurons in *Y* effectively projecting to *X*. Considering that a single neuron of *Y* may contact a single neuron of *X* with multiple synapses (the average redundancy number *ρ*, Figure 1b), we finally connect $\nu_{Y\to X}/\rho_{Y\to X}$ neurons drawn from population *Y* to each neuron of *X* (the non‐integer part of the value is used as a probability of adding one more connection). Depending on the focused or diffuse nature of the projection, these connections are either drawn from the same channel as the one the receiving neuron belongs to or from all channels of the input population.

The dendrites of a neuron are coarsely modelled as a single‐compartment finite cable with sealed‐end boundary condition (Koch, 2005); dendritic attenuation is thus function on three variables: the average diameter of the cylinder model, *d_x_*; the average maximal length of the dendrite, *l_x_*; and the average distance ratio of the dendrite where terminals from population *x* are contacted, *p_x_* (with *p_x_ *= 0 corresponding to contacts on the soma and *p_x_ *= 1 corresponding to contacts on the far end of the dendritic tree). Formally, the electronic constant *L_x_* is defined from these variables and the intracellular resistivity *R_i_* and the membrane resistance *R_m_*:

(5) $$\begin{array}{c} L_{x}\;=\;l_{x}\sqrt{\frac{4}{d_{x}}\frac{R_{i}}{R_{m}}} \end{array}$$

The dendritic attenuation factor$\gamma_{X\leftarrow Y}$ is finally computed as follows:

(6) $$\begin{array}{c} \gamma_{x\leftarrow y}\;=\;\frac{\cosh\left(L_{x}\left(1‐p_{x}\right)\right)}{\cosh\left(L_{x}\right)} \end{array}$$

### 2.2 Plausible parameters from a mean‐field model

In this work, we translate a previously parameterized mean‐field model into a network of leaky integrate‐and‐fire neurons (LIFs). The mean‐field model was optimized to fit of a large set of biological constraints (Liénard & Girard, 2014), and a prerequisite of a successful translation to the integrate‐and‐fire level is the respect of the same constraints. We summarize them briefly in this section and refer readers interested in further details about the genetic multi‐objective evolutionary optimization to the original study.

The first set of biological constraints assesses whether the model is plausible *by construction*, and is thereafter referred to as the *Anatomical objective*. The constraints of this objective validate that the model parameters are within biological plausible ranges. These parameters include axonal bouton counts, dendritic synapses counts and average location of the synapses along the dendritic arborescence. Their optimized values are summarized in Table 2.

**TABLE 2:** Parameter ranges of the integrate‐and‐fire model imported from the mean‐field model

Parameter	Symbol	Optimized range
Bouton number		
MSN → MSN	*α*_(MSN→MSN)_	209–627
FSI → MSN	*α*_(FSI→MSN)_	2,172–4,928
FSI → FSI	*α*_(FSI→FSI)_	26–140
MSN → GPe	*α*_(MSN→GPe)_	171–203
MSN → GPi/SNr	*α*_(MSN→GPi)_	166–288
STN → GPe	*α*_(STN→GPe)_	291–454
STN → GPi/SNr	*α*_(STN→GPi)_	159–239
STN → MSN	*α*_(STN→MSN)_	0–109
STN → FSI	*α*_(STN→FSI)_	0–92
GPe → GPe	*α*_(GPe→GPe)_	37–38
GPe → GPi/SNr	*α*_(GPe→GPi)_	16–17
GPe → STN	*α*_(GPe→STN)_	19–20
CM/Pf → MSN	*α*_(Th→MSN)_	5–15
CM/Pf → FSI	*α*_(Th→FSI)_	2–4
CM/Pf → STN	α_(Th→STN)_	0–1
CM/Pf → GPe	*α*_(Th→GPe)_	0–1
CM/Pf → GPi/SNr	*α*_(Th→GPi)_	0–1
CSN → MSN	*α*_(CSN→MSN)_	248–313
CSN → FSI	*α*_(CSN→FSI)_	4–9
PTN → MSN	α_(PTN→MSN)_	4–9
PTN → FSI	α_(PTN→FSI)_	0–1
Receptor location (% of dendrite length)		
CSN → MSN	*p*_(CSN→MSN)_	80–100
PTN → MSN	*p*_(PTN→MSN)_	76–100
CSN → FSI	*p*_(CSN→FSI)_	80–96
PTN → STN	*p*_(PTN→STN)_	63–100
MSN → MSN	*p*_(MSN→MSN)_	64–88
MSN → GPe	*p*_(MSN→GPe)_	48–60
MSN → GPi/SNr	*p*_(MSN→GPi)_	30–59
FSI → MSN	*p*_(FSI→MSN)_	0–19
STN → GPe	*p*_(STN→GPe)_	23–53
STN → GPi/SNr	*p*_(STN→GPi)_	45–59
GPe → STN	*p*_(GPe→STN)_	28–60
GPe → GPe	*p*_(GPe→GPe)_	0–1
GPe → GPi/SNr	*p*_(GPe→GPi)_	0–15
CM/Pf → MSN	*p*_(Th→MSN)_	26–59
CM/Pf → FSI	*p*_(Th→FSI)_	0–17
Firing threshold (mV)		
MSN	*θ*_MSN_	28–30
FSI	*θ*_FSI_	11–21
STN	*θ*_STN_	24–27
GPe	*θ*_GPe_	6–12
GPi/SNr	*θ*_GPi_	5–7

Note

These ranges correspond to extrema of the solutions optimized in Liénard and Girard (2014). Note that the bouton numbers from basal ganglia afferents (*α*_CSN→*_, *α*_PTN→*_ and *α*_Th→*_) were re‐scaled assuming a pool of 12,000 neurons for each input population.

**FIGURE 2:**
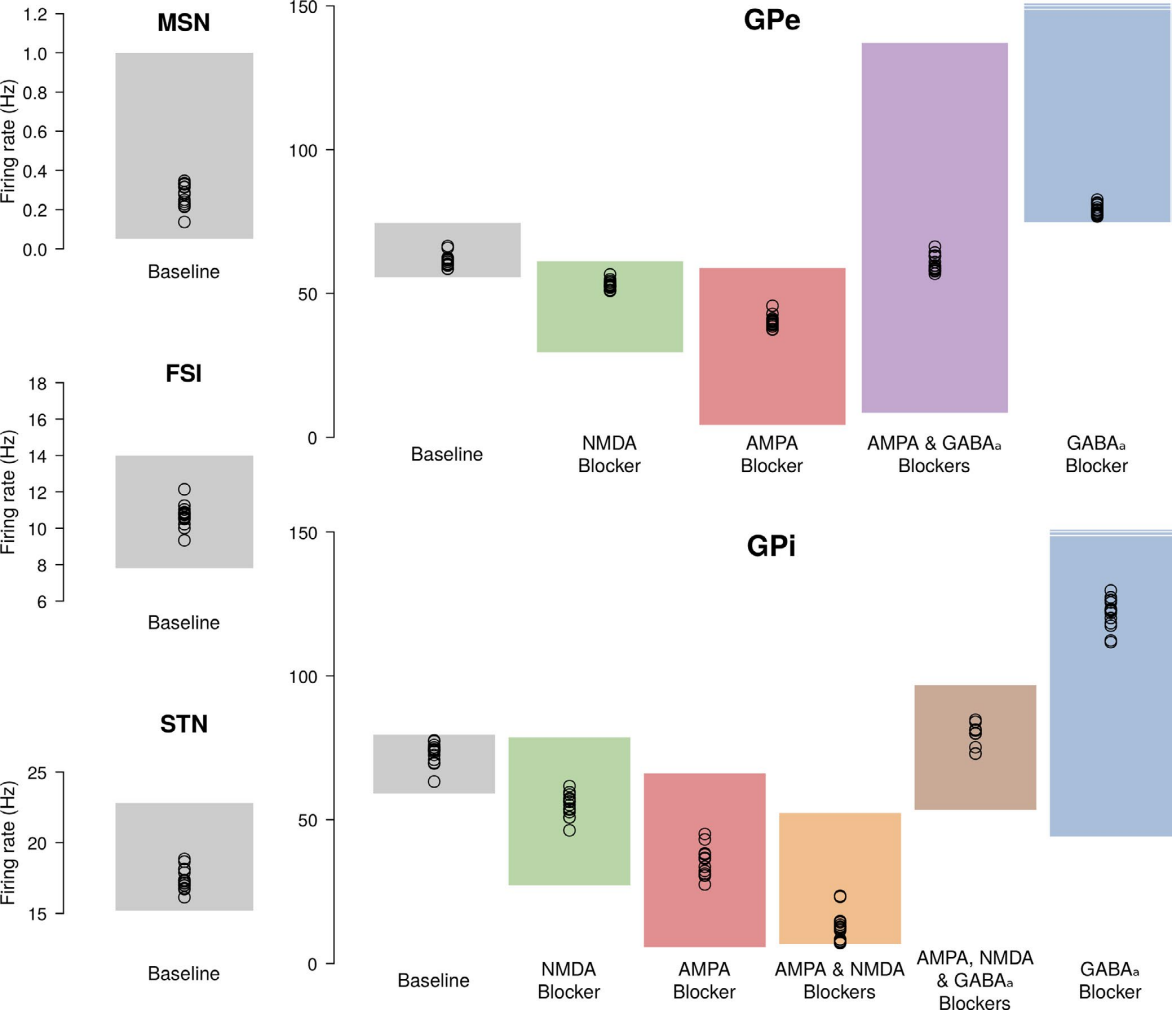
Match between simulated firing rates and biological constraints. The average firing rate of models at rest is shown with circles. The plausible ranges from experimental electrophysiological studies are shown as shaded areas, in grey, for the model in normal condition, and in colours for various pharmacological receptor deactivation tests performed in either the GPe or the GPi (see text for references).

The second set of biological constraints assesses whether the emerging activities of the model appear to be plausible, based on quantitative comparison between simulated and electrophysiological neural recordings. This objective is termed *physiological value objective*. It is realized (a) if each of the simulated firing rates at rest matches a plausible baseline, and (b) if the model can successfully emulate pharmacological deactivation experiments, where the firing rate of neural populations is altered by neurotransmitter blockers. Fourteen such comparisons are made, and the score is incremented by one for each of the verified comparisons, thus defining a 0–14 range of values for this objective. In Liénard and Girard (2014), we established plausible baselines at rest for different nuclei by statistically aggregating activities from 20 different macaque monkeys through a literature survey. We further complement these baselines through the inclusion of more recently published data (Methods section 2.4). The pharmacological experiments are the same as in the previous work and are summarized in Figure 2.

Due to the nature of the *anatomical objective*, there is no need to simulate the model to assess whether it matches these anatomical constraints: assessing the parameters’ range is sufficient. Thus, any theoretically correct translation of plausible parameters at the mean‐field level will be equally correct at the integrate‐and‐fire level. However, to assess whether integrate‐and‐fire models still respect the *physiological value objective*, we need to simulate the neural network and compare its activities against experimental data.

### 2.3 Translation strategy

Most of the integrate‐and‐fire model parameters are directly derived from the mean‐field model parameters (Tables 1 and 2, note that in the latter, each of the fifteen models has one value for each parameter and that we report the min and max of these fifteen values). Three additional degrees of freedom appear when transcribing mean field into spiking models. These properties are the tonic input currents, the refractory periods and, for each connection, the redundancy *ρ* (i.e. the number of synaptic contacts made by one source neuron on a single target neuron). We detail these differences in parameterizations between mean‐field and integrate‐and‐fire models in the following.

First, in the mean‐field approximation, the state of a neuronal population is abstracted as a single value representing the average membrane potential of the neurons composing it (Deco, Jirsa, Robinson, Breakspear, & Friston, 2008). The variability in the post‐synaptic depolarization remains, however, encoded, implicitly, through the use of a sigmoid function coupling the average membrane potential *V* with the firing rate *ϕ*:

(7) $$\phi \left(V\right)\;\;=\;\frac{S_{\max}}{1+\exp\left(\frac{\theta ‐V}{\sigma^{\prime }}\right)}$$

with *S*_max_ being the maximal possible firing rate and *θ* the difference between resting and firing thresholds. The denominator *σ*' can be interpreted as the deviation of firing thresholds of a neural population characterized by an averaged membrane potential (Robinson, Rennie, Rowe, & O’connor., 2004; Van Albada & Robinson, 2009) or as the deviation of individual membrane potentials in a population characterized by a unique firing threshold (Deco et al., 2008).

An important consequence of activities governed by Equation 7 is that the firing rate is always non‐null and reaches zero only as the negative limit of the average membrane potential, $\phi (V)\underset{V\to ‐\infty }{\to }0$. As numerous populations of the basal ganglia exhibit tonic activity even in the absence of inputs, the parameterizations obtained in the mean‐field model made good use of this property of the transfer function of the neuron model to get such tonic activity. In a LIF model, no external input means no spiking activity (the potential *V* tends to *E_r_*, below the firing threshold, see Equation 1), and the only way to have sustained activity in the absence of inputs is to add a positive constant input (*V_C_* in Equation 3). This input has two possible (and not contradictory) interpretations: it can represent additional external inputs that are not explicitly modelled and that are approximated as constant, or internal neuronal excitability properties.

In mean‐field models, neurons from the same population are lumped into a single mass whose state can be described solely by its average membrane potential or, equivalently, its average firing rate (Deco et al., 2008). In Liénard and Girard (2014), weighting this average firing rate, with the average number of synapses *ν* *_Y_*_→_ *_X_*, was sufficient to estimate the inputs sent from population *Y* to the target population *X*. When manipulating models of individual spiking neurons, it becomes crucial to know from how many different neurons these *ν* synapses come from, hence the introduction of the redundancy parameter *ρ* *_Y_*_→_ *_X_* (Figure 1b). It is bounded by a minimal value of 1, when each input synapse comes from a different source neuron (provided that population *Y* contains enough neurons to allow that; otherwise, this minimum will be the size of the population), and a maximal value of *α* *_Y_*_→_ *_X_*, when a single neuron provides all the input synapses. Owing to the difficulty of measuring network dynamics in electrophysiological experiments, and the large density of axonal boutons in the striatum corresponding to MSN, FSI and other interneurons, axonal redundancy is hard to estimate. One notable exception is the electrophysiological study of Koos, Tepper, and Wilson (2004), where three simultaneous post‐synaptic events in one MSN could be attributed to the same spike, meaning that on average each MSN should target each other MSN 3 times (see also Humphries et al., (2010)). In the absence of detailed anatomical data for specific projections in the primate, we opted to generalize this rodent estimate to all connections. We specifically settled on a value of *ρ* = 3 redundant axonal contacts per axon and dendritic field, as a way to approximate the available data. This value has the advantage to fit with plausible orders of magnitude in the striatum, as it results into 70 MSNs targeting one MSN; 39 FSIs targeting one FSI; and 30 FSIs targeting one MSN.

Finally, we set the refractory period of neurons to 2 ms, a value compatible with the maximal firing rates recorded in the basal ganglia (around 400 Hz in the globus pallidus, cf. Nambu et al. (2000), and Wichmann and Soares (2006)), and the membrane time constants with reference to electrophysiological studies or previous modelling efforts: $\tau_{m}^{\text{MSN}}=13$ (Stewart, Bekolay, & Eliasmith, 2012), $\tau_{m}^{\text{FSI}}=16$ (Schulz et al., 2011), $\tau_{m}^{\text{STN}}=26$ (Humphries, Stewart, & Gurney, 2006) and $\tau_{m}^{\text{GPe}}=\tau_{m}^{\text{GPi}}=14$ (Johnson & McIntyre, 2008).

### 2.4 Additional constraint on fast‐spiking interneurons and medium spiny neuron discharge rate

In our previous optimization of mean‐field model parameters (Liénard & Girard, 2014), which are used here in the spiking model, the frequency of FSI was loosely constrained. Indeed, the plausible range of their rest firing rate was [0, 20 Hz], owing to the relative scarcity of electrophysiological recordings of FSI in awake monkeys at the time. As a result, the optimized models displayed highly variable FSI firing rate, from 4.5 to 18.3 Hz (median: 12.2 Hz). In the current work, we rely on recent data that show that FSI, at rest and in macaque monkey, fire at around 10 Hz: 10.1 ± 6.4 Hz, *n* = 36 cells from 2 monkeys (Adler, Katabi, Katabi, Finkes, Prut, & Bergman, 2013); 8.7 ± 2.2 Hz, *n* = 42 cells from 4 monkeys (Yamada et al., 2016); and 12.8 ± 8.9 Hz, *n* = 64 cells from 2 monkeys (Marche & Apicella, 2016). From these and following the same methodology as in Liénard and Girard (2014) to establish confidence intervals from experimental data, we derive the plausible range of FSI discharge rates as [7.8–14 Hz].

Similarly, the MSN constraints in our previous work were also defined in an approximate way, as they were allowed to take on any value in [0, 1 Hz]. However, after the addition of tonic current as a new degree of freedom, it became obvious that the discharge rates of MSN were under‐constrained. In particular, we observed in preliminary works that a large number of parameterizations, setting MSNs to be completely silent at rest, could still lay within the plausible firing rate bounds from Liénard and Girard (2014). These silent parameterizations had highly variable MSN tonic inputs, from as high as 30 mV, and going arbitrarily low (0 mV or any negative value). By contrast, other nuclei display a relatively narrow band of plausible tonic inputs (the width of these ranges being at most 10 mV). To set a more restrictive lower bound on plausible MSN activity, we relied on the electrophysiological study of Adler, Katabi, et al. (2013) which reported firing rates superior to 0.1 Hz most of the time. We note that by design, a truly silent MSN would not be included in such study. Assuming that at least half of the MSNs are not completely silent, we can then assume that the average firing rate of MSN has to be higher than 0.05 Hz. We finally adjust the plausible firing rate range to [0.05, 1 Hz], enabling us to better constrain the range of MSN tonic input current.

### 2.5 Hypersphere solutions

The original parameterizations of Liénard and Girard (2014) can be subsumed into 15 different solutions exhibiting sufficiently different parameters (Liénard et al., 2017). These 15 parameterizations differ in their internuclei connection strengths, resulting in different (but still similar) models that fulfil all the plausibility constraint sets of the *anatomical* and *physiological value* objectives. For each of these parameterizations, after translation to the integrate‐and‐fire level, we varied the tonic input parameter for each neural population (i.e. the 5‐dimensional vector $V_{C}=\left[V_{C_{\text{MSN}}},V_{C_{\text{FSI}}}^{},V_{C_{\text{STN}}},V_{C_{\text{GPe}}},V_{C_{\text{GPi}}}\right]$) on a regular grid. Given a sufficiently fine grid, many combinations of tonic inputs are able to fulfil all plausibility objectives. In order to reduce the number of models investigated and to ensure robustness of firing rate to the inherent model stochasticity (i.e. network wiring and cortical/thalamic spike trains), we sought to determine the most central location within each plausible parameter landscape. In the multidimensional case, this most central location $V_{C}^{0}$ can be defined as the centre of the maximal radius hypersphere fitting within the plausible domain. Noting *F*(*V_C_*) the *physiological value objective* of a parameterization *V_C_*, whose maximal value is 14, we thus look for the solution maximizing the hypersphere radius *r* such as:

(8) $$\forall k\in {\left[‐r,r\right]}^{5},F\left(V_{C}^{0}+r\right)\;=\;14$$

$V_{C}^{0}$ was solved numerically by trying all *k* corresponding to the original grid on which *V_C_* was varied. This was done by maximizing the distance of the 1‐nearest‐neighbour for which $F\left(V_{C_{n}}^{p}+r\right)<14$ on the same grid. For some solution, several candidates for $V_{C}^{0}$ were found, and we broke these ties based on the value closest to the overall median.

### 2.6 Firing rate and power spectra estimations

In all simulations, when population firing rates were to be estimated, the model was first allowed to converge to a stable regime for a duration of one second. Then, the spiking activity of all neurons of the considered population was recorded, and the firing rate was computed as the ratio of the total number of spikes over the number of recorded neurons and the duration of the simulation.

For each model and each neural structure considered, the power spectrum was computed using the discrete fast Fourier transform of the binned (dt = 1 ms) spiking activity of the population during 10‐s‐long simulations, at rest.

### 2.7 Implementation

The code was implemented using PyNEST, the Python bindings of the NEST simulator (Eppler, Helias, Muller, Diesmann, & Gewaltig, 2009), based on version 2.10 of NEST. Individual neurons were simulated using the iaf_alpha_psc_mutisynapse model so as to allow the use of two types of excitatory synapses (to implement the different dynamics of NMDA and AMPA receptors). The model code is available at https://github.com/benoit‐girard/sBCBG.

## 3 RESULTS

### 3.1 Integrate‐and‐fire equivalents of mean‐field models

We obtained leaky integrate‐and‐fire translations maximizing both the anatomical and physiological objectives for each original parameterization of the mean‐field model. This was achieved by (a) setting the redundancy count of axonal boutons from each axon to each dendritic tree to 3, a value compatible with previous estimates (see Methods) and (b) varying the tonic inputs *V_C_* for each nucleus. Optimal tonic levels were overlapping in most neural populations, showing that the different parameterizations of mean‐field models require similar tonic inputs for their translation to integrate‐and‐fire models (Figure S10).

The optimized integrate‐and‐fire models are thus able to match the baseline activity of in vivo, awake monkey recordings at rest (grey areas of Figure 2), as well as mimicking the firing rate changes in response to various antagonist injections (coloured areas of Figure 2). Of interest, two plausible ranges were updated in the current work, compared to Liénard and Girard (2014): the MSN, updated from 0–1 Hz to 0.05–1 Hz to avoid silent models (see Methods), and the FSI, updated from 0–20 Hz to 7.8–14 Hz to reflect more recent experimental data. The MSN of retained solutions were found to have mean firing rates ranging in 0.14–0.35 Hz, showing that (a) completely silent solutions are avoided, and (b) the precise value of the lower bound, here set to 0.05 Hz, is non‐critical as optimized solutions do not reach it in an asymptotic way. The FSI of retained solutions ended up well distributed around the middle of their updated narrower range, showing the overall compatibility of the original parameter set with these new biological data.

Due to the high variability of neuronal activity and the low sample size recorded in neurotransmitter deactivation studies, the plausible ranges are wider than in the baseline activity (Figure 2). Nonetheless, the impact of neurotransmitters is obvious, with opposed effects of glutamatergic transmitters (AMPA and NMDA) and gabaergic transmitter (GABA_A_). In particular, these effects are shown to be cumulative in the simulated model, with simultaneous injections of two or more antagonists resulting in nearly linear summations of their individual effects.

**FIGURE 3:**
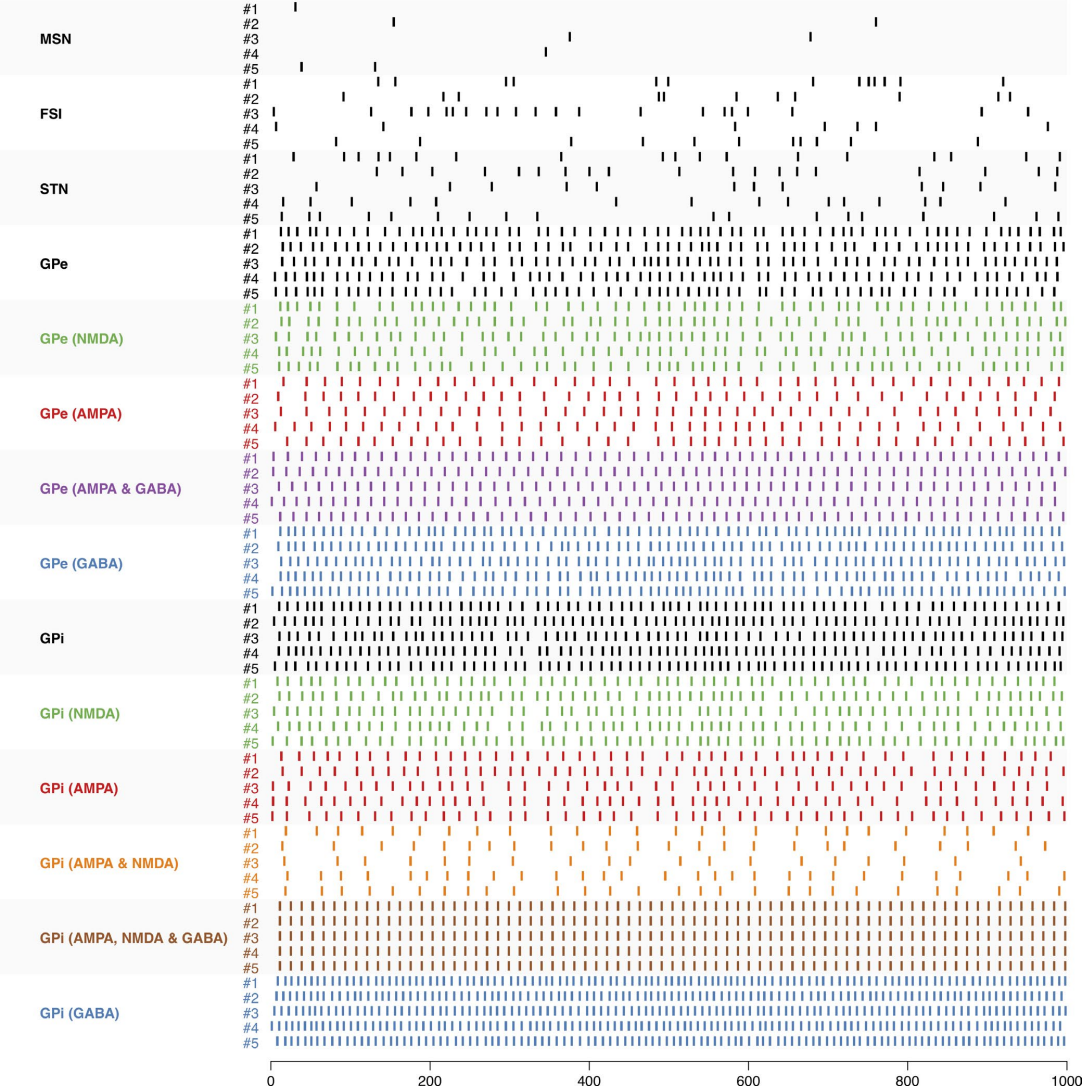
Spike raster during one second obtained for parameterization #9. Traces are shown for 5 neurons of every nucleus at rest (black) and for the nine deactivation studies (same colour code as in Figure 2)

Qualitatively, spike rasters show activity matching the electrophysiological recordings made in non‐human primates (Figure 3). The GPe modelled in our circuit contains only continuously spiking neurons, compatible with the possibility that the pauser neurons observed in vivo belong to a GPe subpopulation with different physiology and connectivity (Mallet et al., 2012). Of interest, the activity becomes highly synchronized when all neurotransmitter blockers are injected in the GPi, with all neurons firing at the same time (brown trace of Figure 3). This matches the observation of Tachibana, Kita, Chiken, Takada, and Nambu (2008) who reported a clock‐like, oscillatory activity in the experimental set‐up.

**FIGURE 4:**
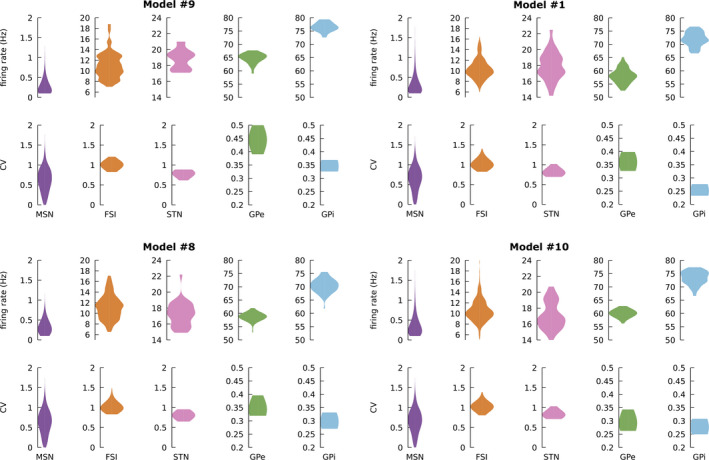
Distributions of firing rates and coefficients of variation (CV) of the neurons of models #9, 1, 8 and 10. 10‐s‐long simulations were used. Concerning the MSNs, neurons that emitted 2 spikes or less were excluded from CV computations (same colour code as in Figure 1)

In addition to the average population firing rates, used as a criterion to validate the model, we computed the distribution of the individual neurons’ firing rates and coefficients of variation (CV) using 10‐s‐long simulations. These distributions do not differ much from one model to another (compare those of models #1, 8, 9 and 10 in Figure 4). For all simulated populations, the firing rate distributions overlap with the core of those measured experimentally (see, e.g., figure 7 of Adler et al. (2012) for MSNs and GPe, figure 2 of Adler, Finkes, Finkes, Katabi, Prut, and Bergman (2013) for MSNs and table 2 of Goldberg, Adler, Bergman, and Fee (2010) for GPe and GPi). The simulated rate distributions also appear narrower than the experimentally observed ones. For example, while most recorded MSNs fire below 1 Hz, a few units can reach 5 Hz. In our different model parameterizations, they are all strictly below 2 Hz. Concerning the CVs, the simulated ones are lower than the experimental ones and also much less widespread (with the extreme case of the GPe and GPi CV distributions). This means that the models fire much more regularly and are also much more homogeneous than the real neural substrate. This limited variability on these two metrics is probably caused, first, by a too homogeneous construction of the models: all cells have the same number of input synapses from the same number of neurons, the same constant input, the same threshold, etc. Adding individual variability around these mean values would probably enlarge the spread of the distributions. The three input populations (CSN, PTN and CM/Pf) are also very homogeneous, as all the Poisson processes of each of these populations have exactly the same average firing rate, rather than a more realistic distribution around these means. The low CVs are also probably caused by the simplistic LIF model we use that lacks internal dynamics that have been documented and modelled (see, e.g., Lindahl and Hellgren Kotaleski (2016); Shouno, Tachibana, Nambu, and Doya (2017)). Fitting these properties was not the goal of this population‐to‐spike translation work, but could be investigated in future work.

**FIGURE 5:**
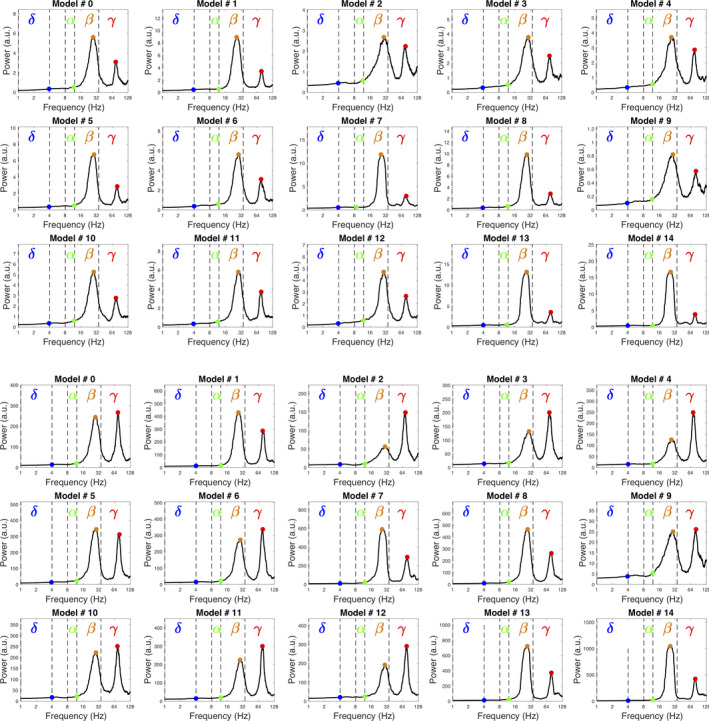
Power spectra of the STN (top) and GPe (bottom) of all models at rest (10‐s‐long simulations). Frequencies are represented on a logarithmic scale. The bands are defined as ranging 2–4 Hz (*δ*), 8–12 Hz (*α*), 12–35 Hz (*β*) and 35–128 Hz (*γ*), respectively. Each dot indicates the maximum value of each band. In the *β* and *γ* bands, these maxima correspond to peaks indicating a true resonant mode in the considered structure

We previously observed (Liénard et al., 2017) that the mean‐field models can start oscillating strongly in the *β* band when slightly perturbed to simulate Parkinson's disease and that these oscillations were generated by the STN‐GPe loop. These oscillations were paroxysmal, as the whole populations of these two nuclei were beating between very low and very high activities in the high‐*β* range. Here, we measured the power spectra of the STN and the GPe of all the fifteen spiking models at rest. In this normal state, while the global activity (Figure 3) does not appear to be dominated by oscillations, the models still systematically exhibit peaks in the *β* range and also in the *γ* range (Figure 5), indicating that they are prone to oscillate in these bands even in resting conditions. As in Liénard et al. (2017), the *β* power results from the delays in the STN‐GPe loop. The *γ* band oscillation is probably linked with the above observation that the GPe (and GPi) CVs are very low: their firing rates (respectively, around 60 and 70Hz) are thus very regular.

### 3.2 Plausible ranges for the different inputs and their effects on the circuit

Our model describes explicitly three sources of inputs to the basal ganglia: two cortical sources, cortico‐striatal neurons (CSN) and pyramidal tract neurons (PTN), as well as centromedian–parafascicular (CM/Pf) thalamic neurons (all the other potential inputs are lumped in the non‐specific constant external inputs, *V_C_*). These inputs are modelled as Poisson noise generators, with one generator per input neuron. How should these external inputs be modulated during activity, for example in an arm‐reaching selection task (Georgopoulos, DeLong, & Crutcher, 1983)? Here, we sought to answer this question by investigating the numbers and firing rates of cortical and thalamic neurons simultaneously activated on competing channels.

We specifically focus on simulating rate‐coded cortical signals, akin to those recorded in arm‐reaching tasks (Georgopoulos et al., 1983). It has been repeatedly noted that in these tasks, discrete populations of cortical neurons code preferentially for one direction, with a rate increase linked to the difference between preferred and actual directions (Georgopoulos, Kalaska, Caminiti, & Massey, 1982; Kalaska, Cohen, Hyde, & M. Prud’Homme., 1989). These neuronal assemblies can further be mapped to cortical columns repeated throughout the motor cortex, possibly reflecting the relevance of having directionally tuned neurons in different behavioural contexts (Georgopoulos, Merchant, Naselaris, & Amirikian, 2007).

The cortical baseline activity of CSN and PTN can be estimated to 2 and 15 Hz, respectively, and the increase in firing rate of individual neurons recruited in arm‐reaching tasks can be further modelled as “baseline + 17.7 Hz” and “baseline + 31.3 Hz” (Bauswein, Fromm, & Preuss, 1989; Turner & DeLong, 2000). It would, however, be an oversight to model the cortical activity in an arm‐reaching task by increasing the cortical activity of all input neurons to a basal ganglia channel. Indeed, given the very large number of cortico‐striatal and cortico‐subthalamic boutons on each dendritic tree, simultaneous increase in cortical firing rate of all afferents would result in tens of thousands additional incoming action potential arriving on each striatal and subthalamic neurons per second. Such input would saturate basal ganglia activity beyond physiological range. Besides, the afferents to a single striatal and subthalamic neuron arise from distinct cortical areas (Draganski et al., 2008; Haber, Kim, Mailly, & Calzavara, 2006; Haynes & Haber, 2013; Lambert et al., 2012), and the extent to which synchronized cortical activity from these areas happens in the course of usual tasks is unclear. Instead, we assume here conservatively that only a fraction of cortical and thalamic afferents is required to elicit downstream activity in the basal ganglia.

**FIGURE 6:**
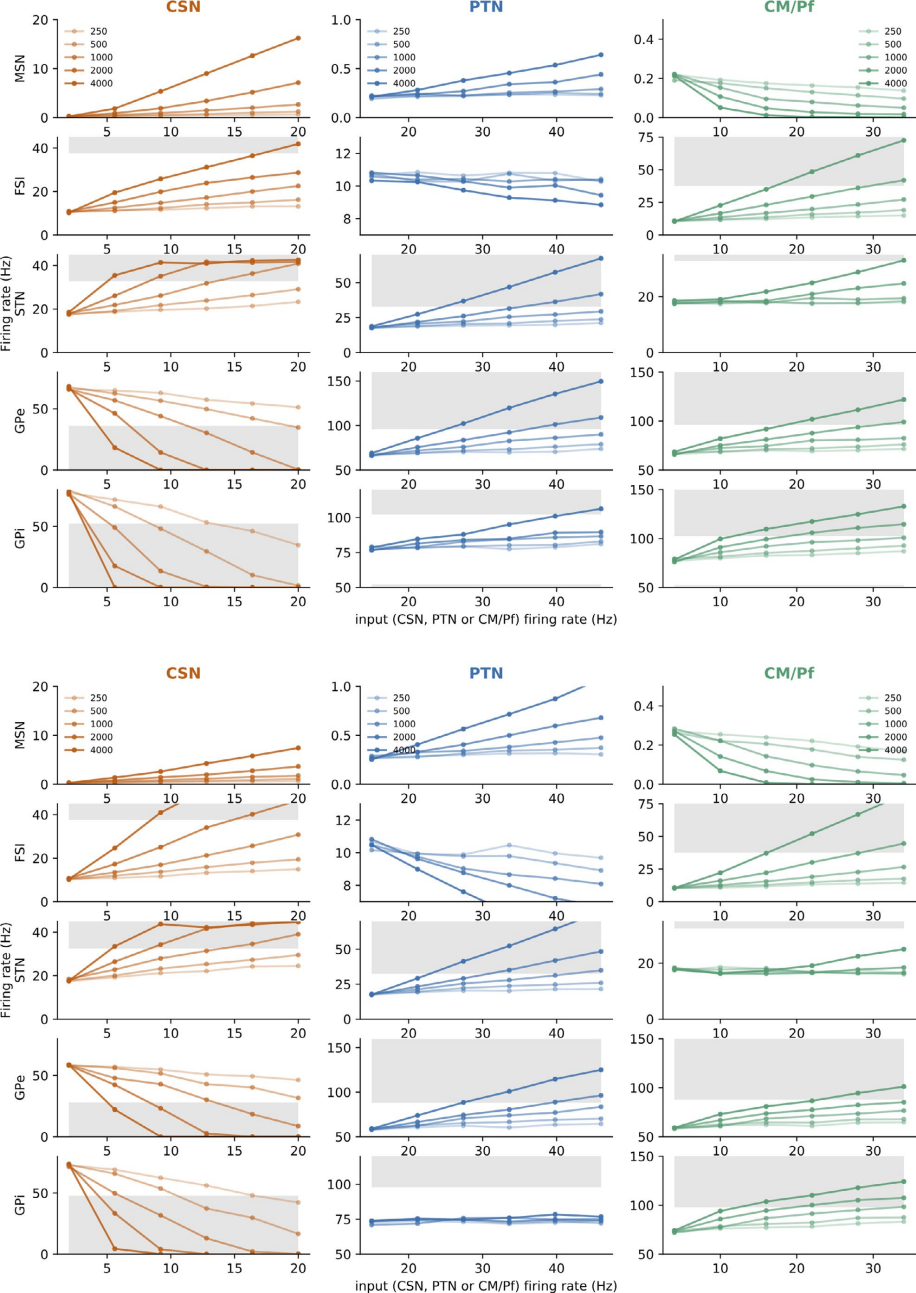
Sensitivity analysis of the basal ganglia model to its inputs (top: model parameterization #9; bottom: model parameterization #1). Each curve represents the evolution of the firing rate of one neural population of the model (MSN, FSI, STN, GPe and GPi, arranged in rows) after stabilization, when the considered input population (CSN, PTN and CM/Pf, in columns) has a given number of neurons activated (200, 500, 1,000, 2,000 or 4,000), with a level of activation varying within ranges representative of the input activities (abscissa, with CSN ∈ [2,20] Hz, PTN ∈ [15, 46] Hz and CM/Pf ∈ [4, 34] Hz). Shaded areas represent implausible levels of activity

As such fraction and its global activity level are unknown, we first sought to investigate the changes in basal ganglia activity as a result of varying the proportion of activated afferents and the amplitude of their activation. For each model parameterization, we tested five activated input population sizes (250, 500, 1,000, 2,000 and 4,000 neurons), whose firing rate we systematically increased from the baseline level (2 Hz for the CSNs, 15 Hz for the PTNs and 4 Hz for the CM/Pf) to levels of high activity (20 Hz for the CSNs, 46 Hz for the PTNs and 34 Hz for the CM/Pf). The variation trends (increase, decrease or absence of significant variations), which fall in five categories, are summarized in Table 3, and the detailed results for models #9 and 1, representative of the two main categories, are reported in Figure 6. Two model parameterizations (model numbers 0 and 12) were excluded from our initial set of fifteen, as they exhibit clearly inadequate (too low) MSN activity under increasing CSN recruitment: even with 4,000 CSN inputs firing at 20 Hz, the average activity of their MSNs remains below 4 Hz.

**TABLE 3:** Sensitivity to input patterns of the various model parameterizations

	#3, 4, 5, 6, 9, 11				#1, 7, 13, 14		
	CSN	PTN	CM/Pf		CSN	PTN	CM/Pf
MSN	↗	↗	↘	MSN	↗	↗	↘
FSI	↗	=	↗	FSI	↗	↘	↗
STN	↗	↗	↗	STN	↗	↗	=
GPe	↘	↗	↗	GPe	↘	↗	↗
GPi	↘	↗	↗	GPi	↘	=	↗

	#10				#2				#8		
	CSN	PTN	CM/Pf		CSN	PTN	CM/Pf		CSN	PTN	CM/Pf
MSN	↗	↗	↘	MSN	↗	↗	↘	MSN	↗	↗	↘
FSI	↗	=	↗	FSI	↗	↘	↗	FSI	↗	↘	↗
STN	↗	↗	=	STN	↗	↗	↗	STN	↗	↗	=
GPe	↘	↗	↗	GPe	↘	↗	↗	GPe	↘	↗	↗
GPi	↘	=	↗	GPi	↘	↘	↗	GPi	↘	↘	↗

The gradual increase in activity and/or recruitment of the input populations, indicated in the columns (CSN, PTN or CM/Pf), causes an increase (↗), a decrease (↘) or an absence of significant variation (=) in the model populations indicated in rows (MSN, FSI, STN, GPe and GPi). We report in orange the patterns that differ from one parameterization to another. The two categories in the top tables comprise most of the models, while each of the bottom table correspond to a single parameterization.

Increasing the numbers and firing rates of activated CSNs leads to similar variation patterns for all parameterizations (Table 3, CSN columns, and Figure 6, left column). It quite naturally results in an increase in the activity of the MSNs and the FSIs, which are directly excited by CSNs. On the other hand, the GPe and GPi that are under strong inhibitory control from the MSNs have a decreasing activity, reaching zero for the largest tested numbers of activated neurons (2,000 and 4,000). This is compatible with the CSN being primarily involved in promoting the selection of their target channel. The activity of STN mirrors the one of GPe and thus converges to a fixed value (around 42 Hz for model #9), when the GPe reaches zero. This is also quite natural: the STN is not directly excited by the CSNs. Therefore, during CSN activity increase, the STN activity increases only because of decreasing GPe inhibition. When this inhibition reaches zero, STN neurons discharge at the maximal rate induced by their tonic potential *V*_STN_.

The PTN inputs yield a different activation pattern (Table 3, PTN columns, and Figure 6, middle column). In the model #9‐like category, as well as for model #10, despite direct projections to the MSNs and the FSIs, the effects on these populations (MSN: small increase, contained under 1 Hz; FSI: small decrease, from 11 to 8 Hz) are limited. The smaller model #1‐like category, as well as models #2 and 8, follows similar increasing/decreasing variations in MSNs/FSIs, except that they are stronger for large numbers of activated input neurons (see Figure 6, lower middle column). For all parameterizations, the STN activity increases notably and drives an increase in the GPe. The effects on the GPi are more diverse: six models react with an increased activity (meaning that the increasing excitatory input from the STN has a stronger effect than the increasing inhibitory input from the GPe, see model #9, Figure 6)), five models exhibit no clear modulation (balanced effects of excitation and inhibition, see model #1, Figure 6, last graph of the middle column), and two models exhibit a decreasing activity (Table 3, models #2 and 8). The first set of models thus supports the idea that the PTN‐driven STN can prevent the selection of a channel, a role commonly attributed to this pathway in models with handcrafted parameterizations (e.g. Gillies and Willshaw (1998); Gurney, Prescott, and Redgrave (2001); Frank (2006); Girard, Tabareau, Pham, Berthoz, and Slotine (2008)).

Finally, the CM/Pf stimulation (Figure 6, right column) results in a significant decrease of activity in MSN and in increasing activity in FSI, GPe and GPi. Concerning the STN, it leads to an increase (Table 3, models #2, 3, 4, 5, 6, 9 and 11, see the representative example of model #9 in Figure 6, upper part) or no modulation, which becomes an increase only for the strongest inputs (Table 3, models #1, 7, 8, 10, 13 and 14, see the representative example of model #1 in Figure 6, lower part). As for GPe and GPi, the result of increasing CM/Pf activity would be hard to guess based on the graph of its projections in the basal ganglia (Figure 1a). Indeed, the CM/Pf projection is diffuse and provides excitatory efferents to the whole basal ganglia, and the many excitatory and inhibitory loops inside the circuit make it impossible to predict the overall influence on GPe and GPi. In all the models studied here, the overall effect of CM/Pf is to control the excitability of the basal ganglia: increased CM/Pf inputs globally prevent selection, by increasing GPi activity at the output level, as well as by decreasing MSN activity and increasing FSI activity at the striatal level. This effect is distinct from the PTN input, which does not really affect the striatum.

The activity levels and/or the number of activated neurons tested here probably extend beyond the normal values in standard task‐related activity. Indeed, we expect activities to reach 30–45 Hz in FSI (Marche & Apicella, 2016), to change by 10–50 Hz in GPe and 10–40 Hz in GPi (Georgopoulos et al., 1983) and 10–20 Hz in STN (Georgopoulos et al., 1983). These plausibility limits are marked by shaded areas in Figure 6, set in the middle of these blurry intervals. As noted above, with large numbers of CSN neurons increasing their activity, the GPe and GPi activity becomes non‐existent, meaning that the corresponding inputs are probably too high. Increasing PTN activity results in STN, GPe and GPi firing rates rising to implausible levels. Also, activating too many CM/Pf neurons simultaneously can result very quickly in very high FSI discharge rates. Based on these observations, in the remainder of our simulations, we will limit the maximal number of input neurons contributing to a given stimulation to 500.

### 3.3 Characterization of action selection

**FIGURE 7:**
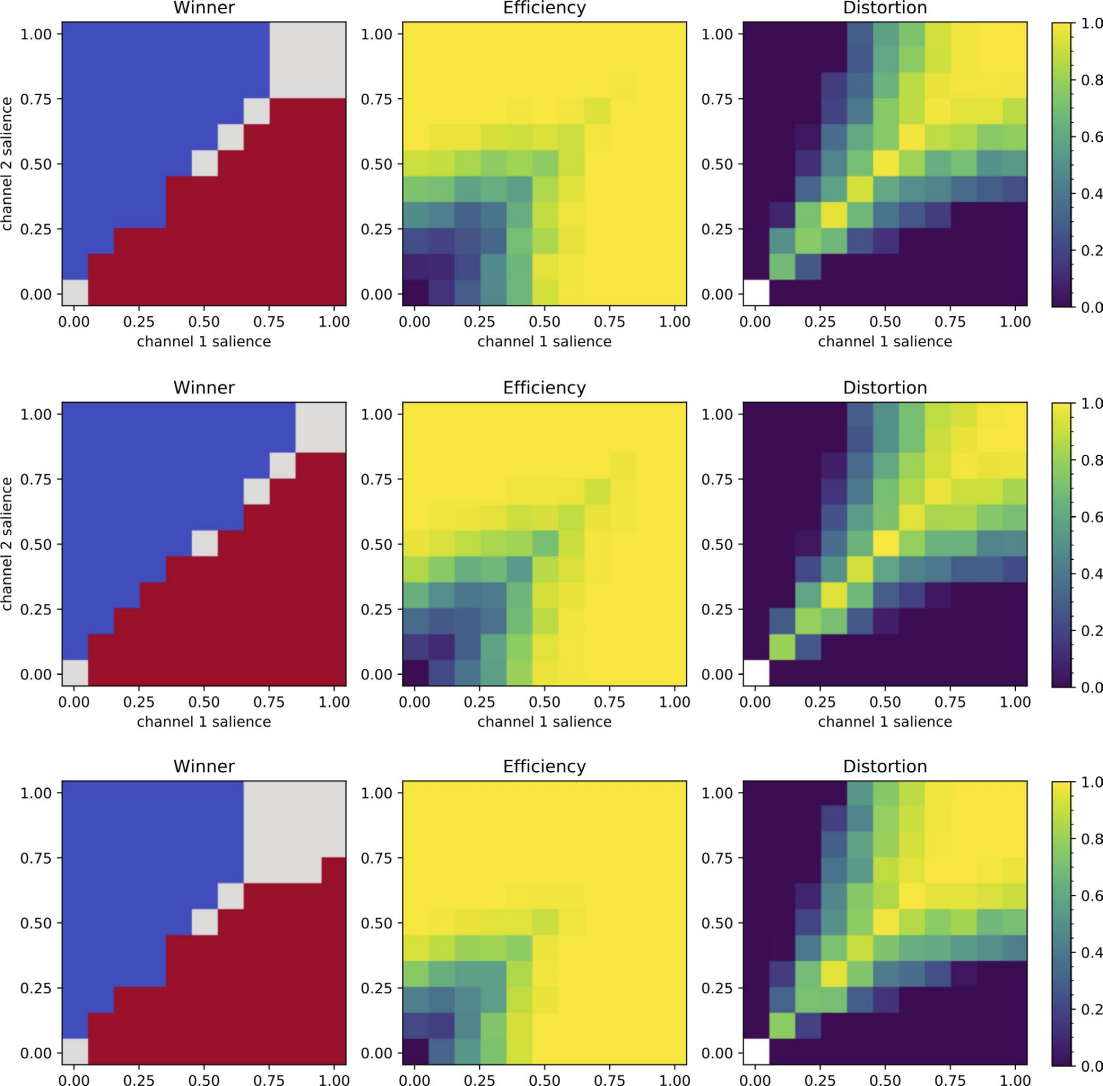
Ability to select competing inputs: Model #9 (top), 1 (middle) and 8 (bottom) action selection properties in the 2‐channel competition task. Left column: winning channel; blue: channel two; red: channel one; grey: both (the difference between the two selection efficiencies is less than 0.1). Middle and Right columns: efficiency and distortion of the selection, as defined in Prescott et al. (2006); see text for further details. White squares in Distortion panels indicate cases where no channels are selected, and thus, distortion is undefined

Following the approach initially proposed by Gurney et al. (2001), and then used by many others (Girard et al., 2008; Humphries et al., 2006; Lindahl & Hellgren Kotaleski, 2016; Prescott, Montes González, Gurney, Humphries, & Redgrave, 2006; Wang, Li, Chen, & Hu, 2007), we characterize action selection by performing a systematic exploration of the input values of two competing channels (Figure 7), while measuring channel disinhibition by the GPi. Following the proposal of Prescott et al. (2006), we use the average GPi firing rate activity to compute the efficiency and distortion of the selection.

The original test gradually increases, from 0 to 1, the input (or salience) of the two channels in competition and measures which channel is the most disinhibited (i.e. selected), how much, and whether its competitor interferes in the selection. The test thus has to be adapted to fit with the three inputs of our model (CSN, PTN and CM/Pf), each of which has its own activation intervals (respectively, 2–20 Hz, 15–46 Hz and 4–34 Hz). Given the results obtained in the previous section concerning the probable role of the CM/Pf to set the global excitability of the circuit, we do not implicate it in the action selection process and therefore keep its input constant during all tests. In the first set of simulations (Figure 7), it is kept at its baseline level of activation (4 Hz), while in the second (Figure 9), three additional levels are tested (5, 6 and 7 Hz). As for the cortical inputs, we randomly select 500 neurons in CSN and in PTN and increase their firing rate linearly from their respective baseline (2 and 15 Hz, respectively) to their maximal rates (20 and 46 Hz, respectively). We record the GPi firing rate $y_{i}^{\text{GPi}}$ of each channel *i*, for each salience input condition, and use it to compute the efficiency, which represents how strongly a channel is selected by computing how much its GPi output is decreased compared to baseline:

(9) $$e_{i}\;=\;\max\;\left(0,1‐y_{i}^{\text{GPi}}/y_{\text{Rest}}^{\text{GPi}}\right)$$

where $y_{\text{Rest}}^{\text{GPi}}$ is the GPi output at rest (reported in Figure 2). The global efficiency *e_w_* of the model for a given input condition is simply the max of the channels’ efficiencies (i.e. the efficiency of the winning channel):

(10) $$e_{w}=\max_{i}\left(e_{i}\right)$$

The distortion represents how much the opponent channel is disinhibited simultaneously to the winning channel and is maximal when both competitors are identically disinhibited:

(11) $$d_{w}\;=\;\frac{\sum_{i}e_{i}‐e_{w}}{\sum_{i}e_{i}}$$

We will add two global measures to these classical ones that we will used to compare the selectivity of models: *e*_Σ_ and *d*_Σ_, respectively, the sum of the efficiencies and of the distortions computed for each salience input condition tested. Therefore, the higher *e*_Σ_ and the lower *d*_Σ_, the better will a given model perform selection without interference between competitors.

Using three‐channel models, we tested all saliences from 0 to 1 with an increment of 0.1. All models exhibited similar selection, efficiency and distortion patterns: this is not trivial, given the variety patterns found in section 3.2 concerning the effect of an increasing activation of the PTNs on the GPi output (increase, decrease or stagnation). We illustrate in Figure 7 the results obtained for models #9, 1 and 8, which exhibited these three different tendencies. They compare quite well with the same metrics measured in previous models parameterized to exhibit action selection (e.g. Prescott et al. (2006), their figure 7 and Girard et al. (2008), their figure 4). Globally, in the models investigated here, the channel with the highest salience is selected (*Winner* panels in Figure 7); low input saliences generate partial selections (*Efficiency* values below one in Figure 7); draw situations, when both channels have identical saliences; tend to result in simultaneous selection (in Figure 7, grey squares on the diagonal on the *Winner* panels when the efficiency difference is below 5%, and even when this difference is larger than that, strong *Distortion* values around the diagonal); finally, high saliences on both channels saturate the system and result in simultaneous selection with maximal distortion. The relatively symmetric arrangement of these measures around the diagonal suggests the system has a relatively stable dynamical behaviour, comparable to Girard et al. (2008) and contrarily to the hysteresis effect exhibited in Prescott et al. (2006). Note, however, that this stability has not been checked analytically and that these previous models included the whole cortico‐baso‐thalamo‐cortical loop, while we are restricted here to the intrinsic basal ganglia circuitry.

It is remarkable that these selectivity properties are the by‐product of an optimization method that was function‐agnostic: the sole purpose of its parameterization was to meet a set of biological constraints and in particular the strength of the connections in the circuit, without making the hypothesis that the basal ganglia should perform action selection (see the definition of the *physiological* objective in section 2.2).

Which parts of the circuit are responsible for this selection capability? Doing a complete analysis of the contribution of each connection of the circuit to selection (using the global efficiency and global distortion metrics) would require to compute their Shapley value (Keinan, Sandbank, Hilgetag, Meilijson, & Ruppin, 2004). Such a computation would unfortunately require measuring efficiency and distortion for all the combinations of connection disruptions. For each connection, many disruptions could be taken into consideration: simply disconnecting it, replacing the input with Poisson generators that have a mean firing rate identical to the rest condition, changing the topology with regard to channel organization, etc. Even if we restrict to one type of disruption per connection only and also only to the connections internal to the circuit (there are 12 such connections), we would still have to test more than 479 million combinations. This is intractable with the time and computational power available to us.

We can, however, have a partial answer to that question by targeting the probable main contributors to selection and measure the effect of their isolated disruptions on the global selection metrics *e*_Σ_ and *d*_Σ_. In classical BG models, three main mechanisms have been proposed to contribute to selection: recurrent lateral inhibitions between MSNs in the striatum, feedforward inhibition of the MSNs by the cortical inputs via the FSIs, and the off‐centre/on‐surround pattern of projections from, respectively, the MSNs and the STN to the GPi and the GPe. We have thus measured *e*_Σ_ and *d*_Σ_ for each model in the intact circuit and with the following four disruptions:

- replacement of the MSN inputs to the MSNs by the same number of Poisson processes, firing at the MSN baseline rate measured at rest,
- replacement of the FSI inputs to the MSNs by the same number of Poisson process inputs, firing at the FSI baseline rate measured at rest,
- replacement of the diffuse STN‐to‐GPi projections by focused ones,
- replacement of the diffuse STN‐to‐GPe projections by focused ones.

**FIGURE 8:**
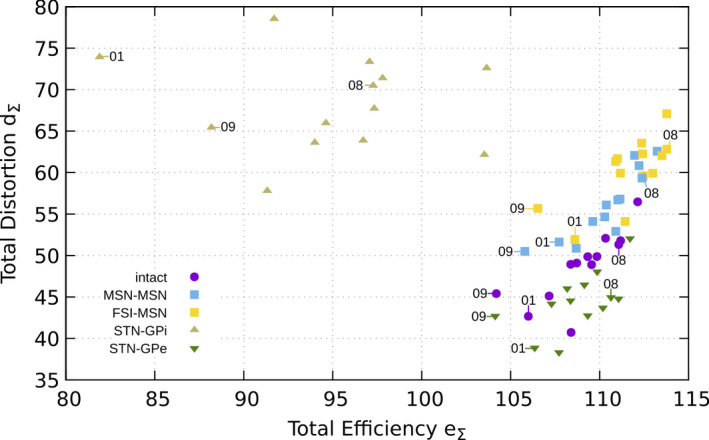
Role of key connections of the circuit in selection: for each model, *e*_Σ_ and *d*_Σ_ are computed for the intact circuit (violet circles), and after neutralization of the MSN‐to‐MSN inhibition (MSN‐MSN, blue squares) and of the FSI‐to‐MSN inhibition (FSI‐MSN, yellow squares), or the transformation of the STN‐to‐GPi (resp. GPe) projections from diffuse to focused (resp. STN‐GPi, upward khaki triangles, and STN‐GPe, downward green triangles), the best selectivity possible is achieved for *e*_Σ_ = 121 and *d*_Σ_ = 0.The points corresponding to models illustrated in other figures are labelled

Simulation results reveal that for all models, the off‐centre/on‐surround pattern of the MSN/STN‐to‐GPi projections is a major component of selection: when transformed into an off‐centre/on‐centre pattern, the ability to select uniformly decreases: *e*_Σ_ strongly decreases and *d*_Σ_ strongly increases (Figure 8, from the intact circuits, violet circles, to the disrupted ones, the khaki upward triangles). Neutralizing either the MSN‐to‐MSN inhibitions or the FSI‐to‐MSN inhibitions (Figure 8, blue and yellow squares) induces a slight increase in the total selectivity *e*_Σ_, that is when selected, channels tend to be more strongly inhibited. However, the distortion is also higher: competing channels are more easily simultaneously selected. These connections thus clearly play a role in setting a good contrast between the winning channel and the others, at the price of a small efficiency decrease. The contribution of the MSN/STN‐to‐GPe projection pattern is the most intriguing one (Figure 8, from violet circles to dark green downward triangles): changing it from a diffuse to a focused projection seems to better the selection compared to intact models. Indeed, this alteration does not really affect total efficiency, but clearly reduces total distortion. Investigating this further is beyond the scope of this study.

**FIGURE 9:**
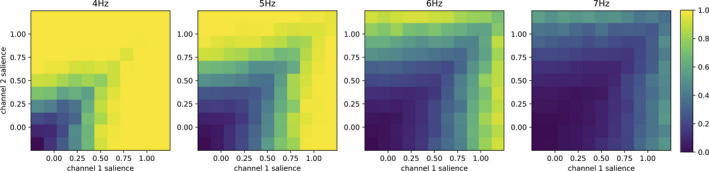
Role of the CM/Pf inputs: Evolution of the efficiency of the selection of model #9 with increasing CM/Pf activity (from left to right: 4, 5, 6 and 7 Hz)

Given the similarity of these action selection metrics across models, despite their differences concerning the effect on the GPi of the activation of the PTNs only, we use model #9 only to further investigate the effect of varying the global and constant CM/Pf input during the selection test. As suggested by the results obtained in section 3.2, increasing the CM/Pf reduced the ability of the circuit to select (Figure 9): the area for which selection does not happen (i.e. where the efficiency is low) increases very rapidly with an increasing CM/Pf input. Thus, we predict that the CM/Pf input controls the selectivity of the basal ganglia. In the context of accumulation‐to‐threshold decision‐making mechanisms implying the basal ganglia (Yartsev, Hanks, Yoon, & Brody, 2018), following the suggestion of Thurat, N’Guyen, and Girard (2015) that the GPi level of inhibition would control the rate of accumulation in the targeted brain regions during the action selection process, we can hypothesize that these thalamic inputs could participate in controlling the overall responsiveness of the basal ganglia, thus modulating the speed–accuracy trade‐off.

## 4 DISCUSSION

In this paper, we presented a leaky integrate‐and‐fire (LIF) model of the monkey basal ganglia circuitry, derived from a previously designed mean‐field model (Liénard et al., 2017; Liénard & Girard, 2014). This original model had fifteen acceptable parameterizations that were originally obtained by an optimization process constrained so as to comply with extensive anatomical and electrophysiological data, in a function‐agnostic manner (i.e. without any hypotheses about the function of the basal ganglia). Notably, it included anatomical constraints derived from Parent et al. (1995) and Lévesque and Parent (2005) which showed that, in monkeys, the so‐called direct and indirect pathways strongly overlap, questioning the explanatory value of the concept of basal ganglia‐segregated pathways inherited from Albin et al. (1989). The formalism adopted at the mean‐field level allowed for a relatively easy conversion to the LIF one, with the addition of five parameters only. We showed here we could derive fifteen LIF models from the fifteen mean‐field ones that comply with the same set of constraints (Figures 2 and 3). We then studied the response of the resulting models to realistic activity increases in the three basal ganglia input sources (cortico‐striatal neurons, pyramidal tract neurons and centromedian/parafascicular thalamic neurons). This helped identifying two parameterizations to be rejected, as their MSNs did not react realistically to vigorous CSN stimulations. This also revealed that the activation of small proportions of these input populations was sufficient to deeply affect the circuit, that the three inputs affected the circuit in radically different manners, that combined CSN and PTN activity increases should be able to elicit selection among competing channels, as proposed in many previous function‐driven basal ganglia models, and finally that the widespread CM/Pf inputs control the excitability of the whole circuit (Figure 6 and Table 3). Finally, we tested a posteriori systematically the ability of the remaining thirteen model parameterizations to perform selection among competing channels. While the models were parameterized in a function‐agnostic manner, all of them appeared to be suitable to perform action selection (Figure 7), showing that the segregation between direct and indirect pathways is not at all necessary to exhibit such a behaviour. Using targeted circuit disruptions, we showed that the off‐centre/on‐surround organization of the MSN/STN inputs on the GPi is indeed a central actor of the selection and that the feedforward and lateral inhibitions in the striatum participate in avoiding simultaneous multiple selections (Figure 8). A final test also confirmed that the CM/Pf input could control the selectivity of the whole circuit (Figure 9) and thus act as a regulator of the speed–accuracy trade‐off. All the tests presented here were done in an average firing rate logic: first, to check that the model passes the same tests as the mean‐field one, and second, to compare to classical action selection metrics, also used in a spiking context (Humphries et al., 2006; Lindahl & Hellgren Kotaleski, 2016). The study of fine temporal dynamics of action selection that cannot be properly studied with mean‐field models will be the object of future investigations on our spiking basal ganglia model.

### 4.1 Robustness of the parameterization

The exploration of varying tonic inputs on the average firing rate of the various nuclei (section 3.1) showed that a large spectrum of values results in plausible firing rates. Here, in order to avoid ending up with tens of LIF model for each of the initial fifteen mean‐field models, we kept only the centre hypersphere parameterization (section 2.5). The tonic input parameters thus have to be in defined ranges, but are not tied to a single brittle configuration.

Fixing the number of synapses that an individual projection neuron makes onto each targeted neurons is clearly a limitation of our model. This information is not required at the mean‐field level, as all the reasoning is made at the average level (average number of synapses coming from one population to each neuron of a target population) and thus does not appear in the original model (Liénard & Girard, 2014). It is, however, necessary at the individual neuron level of modelling, and the current lack of anatomical data providing that level of detail (studies report how many synapses one neuron provides in a given nucleus, but cannot tell how many different neurons in that structure are targeted) forced us to adopt such a bold approach. The choice of a value of three everywhere in the circuit is arbitrary: as stated in section 2.3, it derives from a rat study dealing with MSN‐to‐MSN projections only. It is a compromise between individual input neurons having too low influence on the spiking behaviour of their targets and too much influence resulting in strong firing synchronizations. We checked the sensitivity of this parameter by setting neighbouring redundancy values of 2.75 or 3.25. These resulted in the previously optimized hypersphere parameterizations still achieving perfect or near‐perfect scores (for r = 3.25, there were *n* = 7 parameterizations with a score of 14/14 and *n* = 8 parameterizations with a score of 13/14; for r = 2.75, *n* = 10 parameterization with a score of 14/14, *n* = 4 parameterization with a score of 13/14 and *n* = 1 parameterization with a score of 12/14). Of course, future results that we will obtain with this model when studying its dynamics (oscillations, synchronizations, etc.) may be impacted by that choice, and it is thus desirable to develop experimental anatomical methods that provide that sort of information that we could use to refine the model.

### 4.2 The mean field to LIF parameterization method

As stated in introduction, most basal ganglia modellers, including those designing spiking models (Bahuguna, Aertsen, & Kumar, 2015; Baladron & Hamker, 2015; Berthet et al., 2016; Caligiore et al., 2019; Humphries et al., 2006; Mandali et al., 2015; Stewart et al., 2012; Thibeault & Srinivasa, 2013), handcraft most of the projection weight parameters from one neural population to another with the goal of exhibiting the desired function (in most cases: action selection). The exceptions usually concern a few projections whose weight can be adjusted by learning, like the cortico‐basal projections. The adjustment process that results in these handcrafted weights is not always detailed and often relies on the modeller's inspiration or luck. It is on the other hand trivial to state that in a neural network, the values of the connection weights play an essential role in the behaviour of the model, especially with a heavily recurrent one, like the basal ganglia. Therefore, trying to ground these critical parameters in experimental data is essential if we want to be sure that the fascinating computational objects we design and observe teach us something valuable about the real neural substrate.

Doing extensive parameter search or optimization with large spiking neural networks comprising a few tens of parameters is difficult because of the associated computational cost, which becomes prohibitive when millions of parameterizations of the model have to be simulated and evaluated. The approach we advocate in the ensemble formed by the present and former works (Liénard et al., 2017; Liénard & Girard, 2014) is to first use a detailed but still far less costly mean‐field model to perform parameter optimization aimed at maximizing biological plausibility and then to translate this model into a spiking one. In our case, the optimization was performed with an evolutionary algorithm; 1,000 different runs were carried out with population sizes of 400 and duration of 1,500 generations, thus representing a total of 600 million model simulations. It permitted to find reasonable values for about 50 parameters, while the translation to LIF required the adjustment of five additional parameters (the constant inputs to the neurons in each modelled population) and the choice to have each connection between two neurons relying on three synapses. In order to avoid putting too much a priori hypotheses about the function of the circuit, we suggest to rely as much as possible on anatomical data and on function‐independent measurements and to test a posteriori the operation of the model, possibly using tests driven by hypotheses on the function at that step of the process. For example, while Chersi, Mirolli, Pezzulo, and Baldassarre (2013) used an automated fitting procedure to determine the synaptic weights of their model, they optimized the model with both function‐agnostic constraints (firing rates at rest, as we did) and function‐based ones (action selection behaviour). As such, they could not test the initial hypothesis that the basal ganglia circuit performs action selection.

### 4.3 Mean field to spiking model translation

Mean‐field models were originally designed and applied to the modelling of dynamical patterns in the cortex (Amari, 1977; Nunez, 1974), thalamus (Lopes da Silva, Hoeks, Smits, & Zetterberg, 1974) or at their junction (Wilson & Cowan, 1973). Their application to model the basal ganglia is relatively recent (Tsirogiannis, Tagaris, Sakas, & Nikita, 2010; Van Albada, Gray, Drysdale, & Robinson, 2009). Some of the assumptions developed for the mean‐field formalism take their root in the structure of cortex and thalamus. In particular, mean‐field models rely on the assumption that an arbitrarily large number of neurons with a sustained firing rate can be lumped together, as to study their average activity meaningfully. These models also require that all inputs to a neuron are statistically independent. While these assumptions are well suited to cortical or thalamic populations, their extension to the basal ganglia appears to be less straightforward. Indeed, the medium spiny neurons of the striatum have a very low firing rate, specifically here at rest their activity is in the range of 0.2–0.25 Hz (i.e. once every 4–5 s). Given such sparse individual activity, the law of large number may not apply, and the relevance and meaning of averaging their whole activity with the mean‐field formalism as a whole become problematic. Furthermore, the striatum concentrates more than 98% of the basal ganglia neurons, leaving only 0.3% of them in the STN, 1% in the GPe and 0.5% in the GPi. With such a high dimensionality reduction, there is necessarily a large convergence of the striatal efferences onto the downstream nuclei, at odds with the mean‐field assumption of independent inputs.

In this work, we show that despite these theoretical issues, it is still possible to translate a mean‐field model of the basal ganglia down to the single‐neuron scale—provided that some adjustments are made. We show in particular how the settings of two new degrees of freedom offered in the integrate‐and‐fire network models, namely the tonic currents and the degree of axonal convergence, can be enough to obtain spiking models whose aggregated activity matches one of the mean‐field models.

## CONFLICT OF INTEREST

Authors have no competing interests.

## AUTHORS’ CONTRIBUTIONS

BG and KD designed the research. BG and JL designed and implemented the model. BG, JL and CG ran the simulations. BG, JL and BD analysed them. BG, JL, CG, BD and KD wrote the paper.

## Supporting information

Supplementary Material

Fig S10

## Data Availability

The model code is available at https://github.com/benoit‐girard/sBCBG. The data used to generate the figures of this paper are part of the paper and available as "Data Files" archived on figshare.
