# Supplementary material for: A biologically constrained spiking neural network model of the primate basal ganglia with overlapping pathways exhibits action selection
Source: Eur J Neurosci. 2020 Jul 3;53(7):2254–77. doi: 10.1111/ejn.14869 (PMC8246891; doi:10.1111/ejn.14869)
Supplement: Supplementary file 1 — Supplementary Material [file EJN-53-2254-s002.docx]

Supplementary Materials

Translation from mean-field to integrate-and-fire model

Fig. S10 shows the impact of varying the tonic currents, a newly introduced degree-of-freedom in the building of the spiking model, on the plausibility score defined in Methods. Not surprisingly, the parameter landscapes of the tonic currents reveals the presence of several trade-offs between tonic inputs (Fig. S10). For example, GPi tonic current has to be increased when GPe tonic current is increased, which is also explained by the inhibitory connection from GPe to GPi.

While all optimal tonic currents are found in the same range, the plausible domain of V_FSI_ appear disjointed in the parameter landscape. This difference across parameterizations likely results from the narrower constraint on the FSI firing rate used in this work, which was refined from 0–20Hz to 7.8–14Hz: the FSI tonic current then simultaneously put the FSI firing rates into the new plausible boundaries as well as fixing the mean-field sigmoidal activity function (cf. Methods).

Interestingly, the tonic input required in MSN neurons is consistently larger than in the other neural populations (in the 22-26 mV range for MSN, whereas most other nuclei are in the 5-15 mV range). These higher tonic inputs result in fact from the additional constraint on having non-null firing rate (cf. Methods), as solutions with perfectly silent MSN but fulfilling all the plausible firing discharge are found when decreasing the tonic input current down to arbitrary levels.


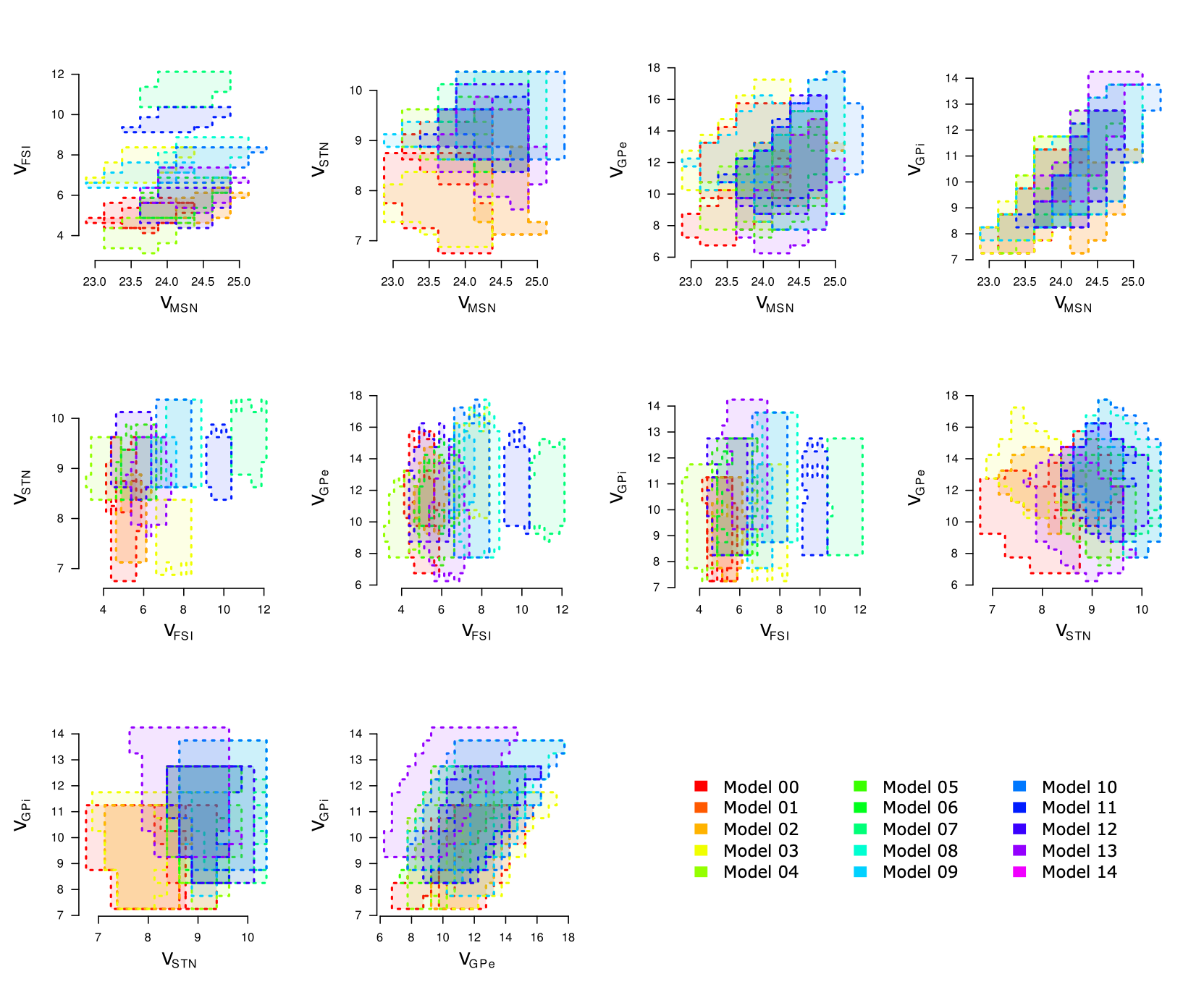


**Figure S10:** Acceptable $V_{C}$ ranges per nuclei: These graphs show the distribution of $V_{C}$ values for MSN, FSI, STN, GPe and GPi that max out the physiological objective. $V_{C}$ parameters were varied systematically over a grid in each of the five dimensions ($V_{MSN}$, $V_{FSI}$, $V_{STN}$, $V_{GPe}$, $V_{GPi}$), and the physiological objective of each solution was assessed. We represent here the all the possible projections of this 5D space on 2D planes, where colored regions represent, for each model, couples of $V_{C}$ values for which a configuration of the three remaining $V_{C}$ allows for maximization of the physiological objective.
