# Supplementary figures and images for: A biologically constrained spiking neural network model of the primate basal ganglia with overlapping pathways exhibits action selection
Source: Eur J Neurosci. 2020 Jul 3;53(7):2254–77. doi: 10.1111/ejn.14869 (PMC8246891; doi:10.1111/ejn.14869)

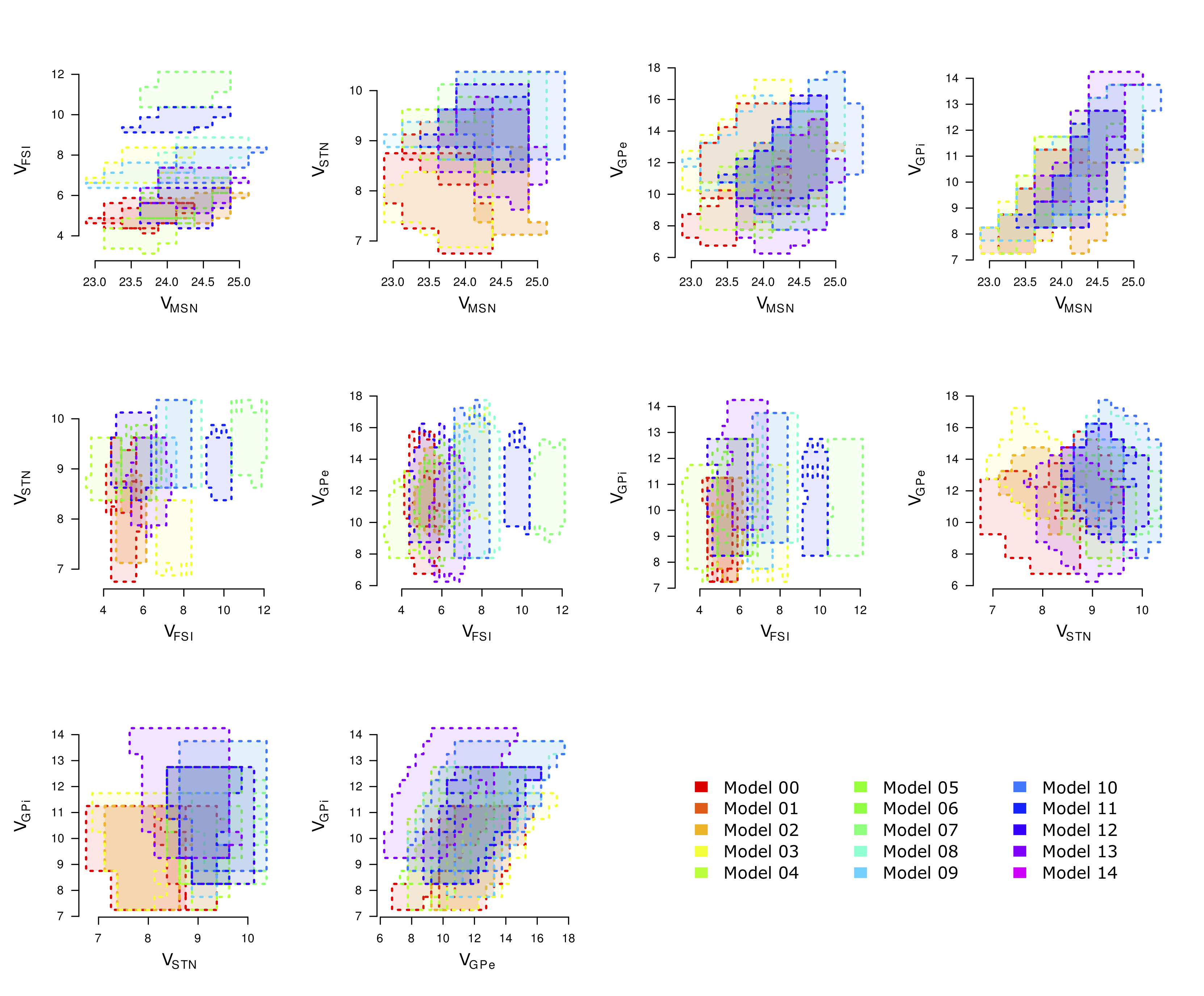

Supplement: Supplementary file 2 — Fig S10 [file EJN-53-2254-s001.png]
